# The Impact of Family Life and Marital Status on the Motivations of Ultramarathoners: The Karkonosze Winter Ultramarathon Case Study

**DOI:** 10.3390/ijerph17186596

**Published:** 2020-09-10

**Authors:** Ewa Malchrowicz-Mośko, Zbigniew Waśkiewicz

**Affiliations:** 1 Faculty of Sport Sciences, Eugeniusz Piasecki University of Physical Education in Poznań, 61-871 Poznań, Poland; 2 Faculty of Sport Sciences, Jerzy Kukuczka University of Physical Education in Katowice, 40-065 Poznań, Poland; z.waskiewicz@awf.katowice.pl

**Keywords:** long-distance running, ultramarathon, motivation, family life, marital status

## Abstract

The aim of this study was to investigate how family life and marital status influence the motivations of ultramarathoners. Two-hundred-sixty-seven Polish athletes participated in the diagnostic survey during the Karkonosze Winter Ultramarathon. The Polish version of the Motivation of Marathoner Scale was used. The motivations of ultra-runners were investigated among the following groups of respondents: people in a relationship/marriage; singles; people with children vs. childless people; people who run alone and with a partner; people who parted with a partner because of lack of support for running. Research results can be useful for sport managers–e.g., findings show the relative importance of modern sport for singles in the second half of life. Research has also shown how people in relationships and people with children can be realized through demanding long-distance running. We also found that running ultramarathons can pose a threat to a relationship.

## 1. Introduction

Ultramarathon running has become increasingly popular among runners’ societies. The number of people who finished an ultra-marathon race in 2016 was 276,535 an amount that has doubled since 2011. Compared to 1.3 million that have run a marathon, 198,000 have run an Ultra Marathon [1]. For long-distance runners, completing an ultramarathon means not only participation in a running event, but above all, a long-lasting physical and mental preparation process. The decision to participate in an ultramarathon usually means entering the subculture of runners and adopting a new social identity, which is very important for ultra-marathoners [2]. Participation in long-distance running meets the criteria of serious leisure [3], not temporary entertainment. Long-distance runners spend a lot of time learning the required running techniques, taking care of their own health (training, sleep, nutrition), traveling to sporting events, and consulting with more experienced runners (while using sports jargon that is often incomprehensible to people who do not run) [4]. To participate in the runners’ subculture, they often have to significantly modify their current lifestyle, which can have a significant impact on their partner and family relationships [1,5].

Recent studies show that ultramarathon runners have higher scores in affiliation compared to runners of shorter race distances [6]. Atkinson studied the behavior of Canadian long-distance triathletes and found that they form a community of pain and together learn to relish physical and mental suffering in sport [7]. Lev suggested that the large audience in front of which the runners perform serves as a stage for gaining social recognition by instrumentalizing their pain in order to strengthen and solidify their running identity [8]. Although the growing interest in participation in ultramarathons among amateurs has become a significant socio-cultural phenomenon (the motivations of ultra-runners were analyzed, among others due to age, gender, running experience, or nationality aspects [9,10,11,12]), little has been written about the family life and marital status of ultramarathoners and the relationship between these aspects with the motives of runners.

Current studies on the relationship between partnership and family life with physical activity and sport generally constitute a research niche. So far, however, it has been established that partner and family relationships can either facilitate or impede participation in and enjoyment of leisure time. Claxton and Perry-Jenkins indicate that leisure time is integral to well-functioning marriages [13]. Barrell et al. have determined that women often do not have the same opportunities to engage in running as men. The leisure time of many women is limited by social expectations. In addition, men often claim that they have the right to rest, saying that they have greater physical, mental, and sporting needs to meet, simply because they are men [14]. Also, according to Janke et al., most widows also reduce their involvement in recreational activities after the death of their spouse [15]. Goodsell and Harris studied the relationships between family life and marathon participation. These authors found that if there is a runner in the family, it can be beneficial for the family, because he/she can thus encourage other family members to lead an active lifestyle, eat healthily, and travel with him/her to discover new places where mass runs are organized. In their opinion, families of runners support them more often than inhibit them. Also, according to researchers, running without the support of a spouse violates the functioning of the marriage because spouses should try to respect their desires [16]. Baldwin et al. showed that with increased support for running in a relationship, marital satisfaction also increases, and runners who ran with a partner experienced a very high level of satisfaction with their marital life [17]. However, Andreasson et al. found that preparations for participating in demanding triathlon competitions disrupt family life [18]. Lamont et al. investigated the relationships of Australian triathletes. They found that wives endured an athlete’s frequent absenteeism, which evoked a range of interrelated stressors. Stressors, however, were counteracted by an array of perceived benefits, such as enriched family lives [19]. Fletcher added that families provide the primary context for their personal leisure experience [20] and Green et al. emphasized the special role of parents’ involvement in encouraging children to participate in sport [21].

However, Lev and Zach claim that even if one partner does not run, but supports the marathon-running partner, adopting a marathon identity might threaten the relationship/marriage if the partner who is not running does not become immersed in this community of runners [22]. In the case of ultra-runners, the situation can be even more complicated and dangerous for the relationship or the functioning of the family, because compared to a marathon, an ultramarathon means even more time spent outside of the training and sports competitions.

It seems important to determine how the ultramarathoners’ motivation might be influenced by family life and marital status. Extremely long training sessions and competitions might “virtually dominate the lives of participants” and consequently their families [23]. Overly extensive sports activities might lead to great “family disharmony” when people prioritize training and competition above the needs of their families [24]. The ultramarathoners’ training might build degenerative tensions between athlete and family in relation to “the resource-intensive nature of endurance sports” [25] because of its time-consuming training [8]. Family life and spousal support (or lack) is an extremely important constraint of competition and practice preparation [18]. During practice and competition, athletes create mental space where the humans’ mind and body try to act as unity to reach the finish line. The influence of spouses plays a dominant role in whether the athlete can have a clear mind or must deal with any inconveniences originating from family life. The results show that having other family members affiliated to the same club and/or participating in the same sporting activity, affects not only sport motivations and involvement, but also the athletes’ level of expertise. Family members are prone to inculcate habits and values, but also to support, help, and encourage relatives in sporting activities [26]. It was stated by many researchers that families have a strong impact on the sport expertise of their spouse. Collins and Buller [27] showed that family has a basic role in the athlete’s development, while Kay [28] determined that elite athletes growing-up in sport and athletes’ originated families. Roderick [29] found that athletes in the family or even somebody practicing sport is an important determinant influencing sports performance and career development. Secure attachment relationships serve as stress and arousal regulator [30]. It creates some kind of mental confidence, which might lead to the difference between success and failure in sports competitions.

Understanding the potential impacts of the participant’s spouse and significant others on ultramarathoners’ motivation is important due to potential flow-on effects across participants’ family and social networks. Despite evidence that an individual’s participation in long-time lasting sports activities can shape the leisure and relationship experiences of families, there is a distinct lack of empirical insights from the perspective of participants and their spouses on motivation to participate in ultramarathons. Specifically, there is a lack of theory surrounding how family status shapes ultrarunners’ motivation.

A widely-known and a recurring theme is how extensive time devotion and mental focus on ultra-marathons can create tension within the family, leading to re-examination, reaffirmation, re-organization, or termination of the relationship, which might consequently lead to changes in an athlete’s motivation [31]. Consequently, this paper responds to the need for a greater understanding of ultramarathoners’ motives by exploring the influence of children or wives/partners on the level of chosen measures of Motivation of Marathoners Scale. It might be hypothesized that spouses of serious ultra-endurance athletes specifically impact their partner’s motivations, thereby contributing to success or failure in the most important competitions.

The aim of this study was an attempt to investigate how family life (having children vs. not having children) and marital status (being in a relationship vs. being single) influence the motivations of ultramarathoners determined with the use of a 56-element scale. We attempted to assess the differences in the level of acquired motivation characteristics, which might lead to a better understanding of the phenomenon being the basic determinant of ultra-marathoners’ successful practice and sports competition.

## 2. Methodology

### 2.1. Method and Research Tool

The study was carried out using the diagnostic survey method during the Karkonosze Winter Ultramarathon. The event was held in March 2020 in Karpacz (Poland).

In the study, the MOMS questionnaire was used (Motivation of Marathoner Scale). The MOMS research tool contains 56 items [32]. This scale shows nine dimensions or specific reasons for running, divided into a broader group of motives:

1. Physical health: general health orientation (six items) and weight concern (four items).
2. Achievement: personal goal achievement (six items) and competition (four items).
3. Social motives: recognition (six items) and affiliation (six items).
4. Psychological motives: psychological coping (nine items), self-esteem (eight items), and life meaning (seven items).

The detailed questions used in the MOMS have been presented in the Appendix A. Answers to items on the MOMS questionnaire are on a seven-point Likert-type scale, where 1 means not a reason, and 7 represents the most important reason. We used the Polish translation of the MOMS questionnaire, adapted and verified for reliability by Dybala [33].

The study was conducted in conformity with the Declaration of Helsinki. As online surveys or questionnaires do not require the completion of a separate participant information sheet or consent form, completion of the survey was deemed to constitute informed consent. Permission was obtained to conduct the study from the event organizers. The survey was anonymous, voluntary and confidential. Respondents were informed about the nature of the survey.

### 2.2. Participants

The study involved 267 ultramarathoners from 391 participants of the 2020 Karkonosze Winter Ultramarathon. The ages of the study participants were distributed as follows: 18–25 years old (five people, 1.8%), 26–35 years old (78 people, 29.2%), 36–50 years old (169 people, 63.2%), and 51–70 years old (15 people, 5.6%). A detailed description was presented in Table 1.

### 2.3. Statistics

The compatibility of the distributions of the scales analyzed with the normal distribution was tested by the Shapiro-Wilk test. Although the distribution was significantly different from normal for some variables, the sample size was large enough that parametric methods were used to verify the hypotheses. When the assumption of homogeneity of variance (tested by Levene’s test) was met, the t-test (Student) for independent variables was used, when the assumption was not met, the Cochran-Cox test was used. Cohen’s coefficient was used as a measure of effect strength. The results when *p*-value < 0.05 were considered statistically significant. Calculations were made in the Statistica 10.0 program (Statsoft; Inc., 2011, Cracow, Poland).

## 3. Results

Investigated ultramarathoners–participants of the Karkonosze Winter Ultramarathon rated running motivations as follows according to MOMS: Health orientation scale (4.71), Self-esteem (4.49), Life meaning (4.43), Psychological coping (4.42), Personal goal achievement (4.22), Affiliation (3.42), Weight concern (3.07), Competition (2.94), and Recognition (2.55).

### 3.1. Runners in Marriage/Relationship vs. Singles

In the first step it was decided to check whether marital status differentiates the motivations of singles and persons in a relationship/marriage (Table 2).

The test result proved to be statistically significant for the Personal goal achievement scale (*p* = 0.043). In this case, the group of people in relationships has higher values. However, the impact of this variable is small. It was determined using the measure of effect strength using the Cohen d coefficient. The calculated value was d = 0.32 (which means small effect). In the case of Health orientation, Competition and Recognition, a result close to the adopted significance limit was obtained, indicating a statistical tendency.

#### 3.1.1. Singles Aged under 35 and Over 35

It was also decided to check if the motives for running differ among young singles (<35 years old) and middle-aged and older (>35 years old) (Table 3).

For Affiliation, the result was statistically significant (higher among singles over 35 years old), and the effect can be interpreted as average (because this interpretation is accepted when the result is in the range of 0.5 to 0.8).

#### 3.1.2. Runners in Marriage/Relationship Aged under 35 and Over 35

The same analysis was carried out for a group of runners in marriage/relationship aged less than 35 years or more than 35 years (Table 4). The result is statistically significant for Personal goal achievement (*p* = 0.004) and competition (*p* = 0.005), being higher for young couples.

### 3.2. Runners Having Children vs. not Having Children

In the next step it was decided to check if people who do not have children present different motivations than people who have children (Table 5).

The study showed no differences between the means in the analyzed groups. In the case of the Competition scale, the result was at the level of statistical tendency (higher for people who have no children).

A similar analysis was carried out only for persons in marriage/relationship (Table 6), which shows runners in Marriage/Relationship Having Children vs. not Having Children. It turned out that the Competition scale is more important for people in marriage/relationship without children (*p* = 0.028).

### 3.3. People Whose Partner Is Running vs. People Whose Partner Is Not Running

At first it was checked if the motives are different among those whose partner is running and among those whose partner is not running (Table 7).

The result turned out to be statistically significant for the Affiliation scale (with small effect strength); higher for running partners.

### 3.4. People Who Parted with their Partner Because of Running

The study showed that among the investigated ultramarathoners, there were nine people who decided to part with their partner because the partner did not run and did not support the passion for running. It was interesting to check what motives are so important to ultra-runners that they are more important than happiness in partnership (Table 8).

The highest values were recorded for Psychological coping (4.22), Health orientation (4.15), and Life meaning (4.19). We also tested how the motivations for running among these people present against the background of runners who never parted with their partner because of running. The result was statistically significant for the Recognition scale (higher in people who did not decide to end the relationship because of running) with a large effect size.

## 4. Discussion

We found that among the investigated ultramarathoners, there were nine people who decided to part with their partner because the partner did not run and did not support their passion for running. The highest values in Psychological coping, Health orientation, and Life Meaning were detected among runners who parted with their partner. These results are in line with the results of previous studies. Crawford et al. indicated that independent leisure might be detrimental to marriage [34]. According to the results of the qualitative study conducted by Lev and Zach [22], married life is susceptible to deterioration or even going through a crisis, given the process of embracing a running identity. Without a partner’s support, runners are unable to continue running marathons. They note there is a thin line that is maintained by the runner, knowing that crossing it would lead to severe marital difficulties. A few marathon runners participating in this Israeli study also decided to divorce. In this research, there were, among others, statements such as [22]: “at some point running become the most important thing in the world, you lose focus on other matters in life… In such a situation, it is very difficult to manage married life because you are absent at all times”; “in the most critical morning hours, you have to prepare the children to go to school, and you are not there. Then you are absent on Fridays and Saturdays, and when you return, you are so tired. In the evening, the time to be with your husband, you are tired, and all you want to do is go to sleep. One of the main problems is that when you finally spend time with your spouse, it’s so boring because both are in different places. You are part of a whole new world that your spouse not only isn’t part of, and, tragically, he finds it boring. A stranger won’t understand this, most of my teammates in the running group also experience it, which helps”; “my husband and I don’t speak the same language anymore, our main interests have become extremely different since I joined the running group”; “I began very intensive training sessions that caused a crisis in our marriage, so to prevent an earthquake, I decided to make her love what I love. I thought that in order to continue, I must incorporate her into the same hobby as mine”; “Despite the fact that my wife and kids were very supportive, we all made compromises. After the marathon event, I understood that I had to slow down, to return home, and compensate them for being absent for such a long time”. However, another marathon runner in this study added: “My husband is amazing. He is so supportive”. Another runner found new love thanks to running: “Finally I found someone who can truly understand me, someone that comes from my world”.

We also checked how the motivations for running among these people present against the background of runners who never parted with their partner because of running. The result is statistically significant for the Recognition scale (higher in people who did not decide to end the relationship because of running) with a large effect size. In fact, it turned out that people who did not take part in their partner by running could count on their support, and at the same time, it was important for them to gain recognition and admiration among loved ones.

Researchers began to examine how different types of leisure affect couples in different ways. Two groupings of leisure have emerged: leisure time spent with one’s spouse and leisure time spent without one’s spouse. It has been investigated how leisure relates to marital satisfaction [13]. In our study, we decided to test if there is a difference in motivations to run among people who run with a partner or alone. The result turned out to be statistically significant for the Affiliation scale–higher for running partners. For runners in a relationship/marriage, the possibility of sharing leisure time with friends, family, and partner was of higher importance. The other motives were of similar importance for both groups of respondents.

Only one statistically significant difference also appeared between the motivations of ultra-runners in the relationship and singles. In the leisure industry (e.g., tourism), there are increasing offers targeted only at singles. However, as it turned out, in the case of sport (ultramarathons), there is no need to prepare very special offers or marketing campaigns targeted at singles because they mostly have the same motivations as ultra-runners in a relationship. The test result proved to be statistically significant for the Personal goal achievement scale–the group of people in relationships has higher values. This might be because singles usually have more free time and thus can prove themselves on many levels of life. Also, for example, a person who runs has decided to give up work for a partner or family, then in the sport, he/she seeks the opportunity to test himself/herself.

However, the interesting issue is that for singles over 35 years old, participation in ultramarathons plays a more important affiliate role than for younger singles (less than 35 years old). This might be because young singles usually have more opportunities than older people to socialize outside work, fewer responsibilities, more opportunities to make new friends or possibly finding a partner. For singles over the age of 35, these functions are fulfilled by sport (running ultramarathons). This issue might be underlined in sports promotion. This issue might be considered when promoting sport among lonely people over 35 years old.

The same analysis was carried out for a group of runners in marriage/relationship aged less than or more than 35 years old. The result was statistically significant for Personal goal achievement (*p* = 0.004) and competition (*p* = 0.005), being higher for young couples. Therefore, it turns out that young couples are more often looking for competition and self-struggle than people over 35 years old in relationships.

Cowan and Cowan reported that childless couples, compared to couples who had children, tended to rate themselves as spending more companionate time together [35]. Kurdek examined couples undergoing the transition to parenthood and a comparison group of nonparents and noted that new parents reported a sharper decline in joint activities than nonparents [36]. We considered it important to examine whether runners who have children have different motivations to participate in ultramarathon than childless people. Also, Goodsell et al. [37] indicated that researchers and those who want to encourage long-term engagement in active leisure should consider family context as an important influence on people’s motivations to run. It turned out that in the case of running an ultramarathon, the motivations differ between runners in marriage/relationship having children and runners in marriage/relationship not having children. We found that the Competition scale is more important for people in marriage/relationship without children.

## 5. Limitations of the Study and Future Lines of the Research

Our study collected information from individuals who attended the ultramarathon in March 2020 in Karpacz, so these athletes do not represent others who used to do ultramarathon, but did not continue or dropped off from this sports event. Any future study should include a long-term survey of individuals who used to participate ultramarathon, but not anymore. We investigated ultramarathoners because mass running events are increasingly popular and stimulate the development of running tourism, but also other demanding sports disciplines should be investigated in the field of marital status and family life of athletes.

## 6. Conclusions

This article analyzes the impact of family and partner relationships on the motivations for participating in ultramarathons. We found that running ultramarathons can pose a threat to a relationship. It would be worthwhile for those who promote and organize running events encourage running together with a partner, because it can be beneficial for the relationship and family. Research results can be useful for sport managers–they show the importance of modern sport, including affiliate for singles in the second half of life. Research has also shown how people in relationships and people with children can be realized through demanding long-distance running.

## Figures and Tables

**Table 1:** Detailed description of ultramarathon runners (*n* = 267).

Variable	Description	
Sex	Men	Women
	204 (76.4%)	63 (23.5%)
Marital status	Single	In relationship
	46 (17.3%)	221 (82.7%)
Children	Yes	No
	173 (47.9%)	94 (35.5%)
Running with partner *	Yes	No
	106 (47.9%)	115 (52.1%)

* 9 runners (3.3%) parted with the partner because the partner did not run and did not support the passion for running.

**Table 2:** Runners in marriage/relationship vs. singles.

MOMS Scales	Runners in Marriage/Relationship (*n* = 221)		Singles (*n* = 46)		t	*p*	d
	M	SD	M	SD			
Health orientation	4.79	1.44	4.30	1.77	1.75	0.085	0.30
Weight concern	3.14	1.66	2.77	1.58	1.37	0.173	0.23
Personal goal achievement	4.29	1.26	3.87	1.41	2.03	0.043	0.32
Competition	3.02	1.64	2.57	1.41	1.72	0.086	0.29
Recognition	2.61	1.41	2.24	1.26	1.66	0.098	0.28
Affiliation	3.40	1.57	3.53	1.70	−0.50	0.621	0.08
Psychological Coping	4.40	1.48	4.53	1.53	−0.54	0.589	0.09
Life Meaning	4.42	1.43	4.46	1.64	−0.17	0.866	0.03
Self-esteem	4.49	1.41	4.48	1.66	0.03	0.978	0.00

**Table 3:** Motivations of singles aged under 35 and over 35.

MOMS Scales	Less Than 35 Years Old (*n* = 22)		More Than 35 Years Old (*n* = 24)		t	*p*	d
	M	SD	M	SD			
Health orientation	4.11	1.65	4.49	1.90	0.72	0.474	0.21
Weight concern	2.72	1.55	2.82	1.63	0.23	0.821	0.07
Personal goal achievement	4.11	1.60	3.64	1.20	−1.15	0.258	0.34
Competition	2.63	1.54	2.52	1.32	−0.25	0.806	0.07
Recognition	2.28	1.36	2.20	1.20	−0.21	0.835	0.06
Affiliation	2.95	1.74	4.05	1.51	2.28	0.028	0.67
Psychological Coping	4.40	1.61	4.65	1.47	0.54	0.594	0.16
Life Meaning	4.23	1.63	4.67	1.67	0.90	0.372	0.27
Self-esteem	4.29	1.67	4.66	1.68	0.74	0.462	0.22

**Table 4:** Motivations of runners in marriage/relationship aged under 35 and over 35.

MOMS Scales	Less Than 35 Years Old (*n* = 61)		More Than 35 Years Old (*n* = 160)		t	*p*	d
	M	SD	M	SD			
Health orientation	4.60	1.35	4.87	1.47	−1.25	0.211	−0.19
Weight concern	2.97	1.64	3.20	1.67	−0.94	0.350	−0.14
Personal goal achievement	4.69	1.24	4.14	1.24	2.94	0.004	0.44
Competition	3.52	1.80	2.83	1.53	2.86	0.005	0.42
Recognition	2.77	1.49	2.55	1.37	1.05	0.293	0.16
Affiliation	3.41	1.53	3.39	1.59	0.09	0.930	0.01
Psychological Coping	4.51	1.38	4.36	1.52	0.68	0.500	0.10
Life Meaning	4.71	1.38	4.31	1.43	1.86	0.065	0.28
Self-esteem	4.76	1.31	4.38	1.44	1.78	0.076	0.27

**Table 5:** Runners having children vs. runners not having children.

MOMS Scales	Runners Having Children (*n* = 173)		Runners Not Having Children (*n* = 94)		t	*p*	d
	M	SD	M	SD			
Health orientation	4.76	1.58	4.62	1.37	0.70	0.487	0.09
Weight concern	3.13	1.66	2.97	1.64	0.77	0.441	0.10
Personal goal achievement	4.14	1.27	4.35	1.33	−1.29	0.197	0.16
Competition	2.80	1.51	3.19	1.74	−1.83	0.069	0.24
Recognition	2.50	1.33	2.63	1.50	−0.71	0.478	0.09
Affiliation	3.49	1.63	3.29	1.52	0.97	0.331	0.13
Psychological Coping	4.40	1.49	4.47	1.48	−0.35	0.726	0.04
Life Meaning	4.36	1.47	4.56	1.45	−1.08	0.281	0.14
Self-esteem	4.40	1.45	4.64	1.45	−1.28	0.201	0.16

**Table 6:** Runners in marriage/relationship having children vs. runners in marriage/relationship not having children.

MOMS Scales	Children: Yes (*n* = 157)		Children: No (*n* = 64)		t	*p*	d
	M	SD	M	SD			
Health orientation	4.81	1.51	4.74	1.25	0.36	0.718	0.06
Weight concern	3.17	1.65	3.05	1.70	0.47	0.640	0.07
Personal goal achievement	4.19	1.27	4.53	1.20	−1.82	0.071	0.27
Competition	2.85	1.52	3.43	1.84	−2.23	0.028	0.35
Recognition	2.54	1.34	2.78	1.57	−1.07	0.287	0.16
Affiliation	3.44	1.61	3.29	1.49	0.62	0.534	0.09
Psychological Coping	4.37	1.49	4.49	1.45	−0.56	0.579	0.08
Life Meaning	4.31	1.45	4.71	1.35	−1.91	0.058	0.29
Self-esteem	4.37	1.42	4.78	1.36	−2.00	0.047	0.30

**Table 7:** Motives of runners who run with partner and without partner.

MOMS Scales	Running Partner (*n* = 106)		Not running Partner (*n* = 115)		t	*p*	d
	M	SD	M	SD			
Health orientation	4.78	1.57	4.64	1.44	0.71	0.478	0.09
Weight concern	3.16	1.75	3.06	1.62	0.45	0.651	0.06
Personal goal achievement	4.35	1.27	4.16	1.28	1.17	0.245	0.15
Competition	3.04	1.62	2.93	1.60	0.51	0.612	0.07
Recognition	2.60	1.34	2.58	1.46	0.06	0.952	0.01
Affiliation	3.67	1.63	3.09	1.51	2.81	0.005	0.37
Psychological Coping	4.44	1.52	4.36	1.48	0.37	0.709	0.05
Life Meaning	4.51	1.47	4.32	1.39	0.99	0.323	0.13
Self-esteem	4.54	1.39	4.42	146	0.64	0.524	0.08

**Table 8:** Motives of runners who parted with partner because of running vs. runners who never parted because of running.

MOMS Scales	Parting by Running: Yes (*n* = 9)		Parting by Running: No (*n* = 228)		t	*p*	d
	M	SD	M	SD			
Health orientation	4.15	2.08	4.65	1.47	0.99	0.323	0.28
Weight concern	3.08	1.91	3.02	1.62	−0.11	0.909	0.04
Personal goal achievement	3.93	1.72	4.23	1.26	0.69	0.489	0.20
Competition	3.06	1.79	2.98	1.62	−0.14	0.885	0.05
Recognition	1.50	0.81	2.65	1.41	4.00	0.002	1.03
Affiliation	2.83	1.58	3.35	1.55	0.97	0.333	0.33
Psychological Coping	4.22	1.67	4.42	1.47	0.40	0.686	0.13
Life Meaning	4.19	1.57	4.42	1.47	0.46	0.648	0.15
Self-esteem	3.75	1.80	4.52	1.40	1.60	0.112	0.48

## Appendix A

**Table A1:** MOMS–MOTIVATIONS OF MARATHONERS SCALE (Possible reasons to run).

Health orientation
To improve my health
To prolong my life
To become more physically fit
To reduce my chance of having a heart attack
To stay in physical condition
To prevent illness
**Weight concern**
To help control my weight
To reduce my weight
To look leaner
To stay physically attractive
**Personal goal achievement**
To improve my running speed
To compete with myself
To try to run faster
To push myself beyond my current limits
To see if i can beat a certain time
To make my body perform better than before
**Competition**
To compete with others
To see how high i can place in races
To get a faster time than my friends
To beat someone I have never beaten before
**Recognition**
To earn respect of peers
To earn the respect of people in general
To make my family or friends proud of me
People look up to me
It brings me recognition
To get compliments from others
**Affiliation**
To socialize with other runners
To have something in common with other people
To meet people
To participate with my family or friends
To share a group identity with other runners
To visit with friends
**Psychological coping**
To become less anxious
To become less depressed
To distract myself from daily worries
To improve my mood
To have time alone to sort things out
To concentrate on my thoughts
To solve problems
To blow off steam
To get away from it all
**Life meaning**
To add a sense of meaning to life
To make my life more purposeful
To make myself feel whole
To make my life more complete
To feel a sense of belonging in nature
To have time alone with the world
To feel at peace with the world
**Self-esteem**
To improve my self-esteem
To feel more confident about myself
To improve my sense of self-worth
It is a positive emotional experience
To feel proud of myself
To feel a sense of achievement
To feel mentally in control of my body
To feel like a winner

Own elaboration; Source: from authors of MOMS [32].
